# Clinical features of central nervous system involvement in patients with eosinophilic granulomatosis with polyangiitis: a retrospective cohort study in China

**DOI:** 10.1186/s13023-021-01780-x

**Published:** 2021-03-31

**Authors:** Suying Liu, Ling Guo, Xiaoyuan Fan, Zhaocui Zhang, Jiaxin Zhou, Xinping Tian, Mengtao Li, Xiaofeng Zeng, Li Wang, Fengchun Zhang

**Affiliations:** 1 Department of Rheumatology and Clinical Immunology, Peking Union Medical College Hospital, Chinese Academy of Medical Sciences and Peking Union Medical College, the Ministry of Education Key Laboratory, National Clinical Research Center for Dermatologic and Immunologic Diseases, Beijing, 100730 China; 2 Department of Rheumatology, Dongying People’s Hospital, Dongying, 257000 Shandong Province China; 3 Department of Radiology, Peking Union Medical College Hospital, Chinese Academy of Medical Sciences and Peking Union Medical College, Beijing, 100730 China; 4 Department of Rheumatology and Clinical Immunology, Gansu Province People’s Hospital, Lanzhou, 730000 Gansu Province China

**Keywords:** Central nervous system involvement, Eosinophilic granulomatosis with polyangiitis, Churg-Strauss, Clinical features, Associated factors

## Abstract

**Background:**

Central nervous system (CNS) involvement is extremely rare in eosinophilic granulomatosis with polyangiitis (EGPA), but is associated with a poor prognosis in the five-factor score. This study aims to elucidate the clinical features and independently associated factors of EGPA with CNS involvement.

**Results:**

CNS involvement was observed in 17.3% (19/110) of EGPA patients from Peking Union Medical College Hospital between 2007 and 2019. We retrospectively reviewed their clinical data and analyzed the independently associated factors. Their mean age was 51.7 ± 11.56 years with no male/female predominance. Ischemic lesions were the most common manifestations, accounting for 63.2% of the 19 cases, followed by posterior reversible encephalopathy syndrome (36.8%), spinal cord involvement (15.8%), medulla oblongata involvement (15.8%), and intracranial hemorrhages (15.8%). Compared to the control group, patients with CNS involvement were of older age (51.7 ± 11.56 vs. 43.7 ± 13.78 years, *p* = 0.019) and had a higher ratio in the digestive tract involvement (52.6% vs. 28.6%, *p* = 0.042). Further multivariate analysis revealed that age, disease duration, and fever were the potential independent risk factors for CNS involvement of EGPA. Glucocorticoids combined with cyclophosphamide were the strategic therapy (94.7%). Intrathecal injections of dexamethasone and methotrexate were administered to 21.1% of the patients. Although seven patients relapsed during glucocorticoid reduction, seventeen patients finally achieved clinical remission. One patient died of acute intracerebral hemorrhage within one month, and another died of gastrointestinal perforation. Outcomes and cumulative survival show no significant differences between the two groups.

**Conclusions:**

CNS involvement is uncommon in EGPA with various manifestations, and ischemic lesions are the most frequent. Age, disease duration, and fever are independent factors associated with CNS involvement in EGPA. The therapy of glucocorticoids combined with cyclophosphamide and intrathecal injections yields favorable responses. Acute intracranial hemorrhage and gastrointestinal perforation may be the principal causes of death.

## Background

Eosinophilic granulomatosis with polyangiitis (EGPA) is a rare systemic necrotizing antineutrophil cytoplasmic antibody (ANCA)-associated vasculitis (AAV). Although EGPA was classified as a type of AAV, in the past few decades, researchers have increasingly considered it as a distinct form of AAV with different manifestations and outcomes as compared to granulomatosis with polyangiitis (GPA) and microscopic polyangiitis (MPA) [1]. ANCA can be detected in approximately one-third of EGPA patients’ serum, and the clinical spectrum and pathogenesis of ANCA-negative and positive EGPA are different [2–4].

EGPA primarily affects the respiratory tract, lungs, peripheral nervous system, heart, gastrointestinal tract, and skin, but central nervous system (CNS) involvement is rarely seen [5–7]. CNS involvement was included in the 1996 five-factor score (FFS) that was associated with a higher risk of death, but it was removed from the 2011 revised FFS system due to its rarity [8, 9]. There are few systematic studies on CNS involvement of EGPA, and most of them are case reports. The largest study at present is a retrospective cohort in France, which included 26 patients with CNS involvement of EGPA [10]. For the Chinese EGPA population with CNS involvement, a comprehensive study has not yet been reported. Therefore, we collected the clinical data of EGPA patients in our hospital, aiming to investigate the clinical features, independently associated factors, treatment, and outcomes.

## Results

### Features of CNS involvement in EGPA

A total of 19 EGPA patients developed CNS involvement in this cohort (17.3%). The mean age of EGPA onset was 51.7 years (range 25–72 years) with no female/male predominance. Table 1 shows a summary of CNS involvement in EGPA, and the specific lesion characteristics for each patient are shown in Table 2.

**Table 1:** Clinical features of CNS involvement in EGPA (n = 19)

	Number	Ratio (%)
*Cerebrovascular events*	14	73.7
Ischemic infarctions	12	85.7
Intracranial hemorrhages	3	21.4
*Posterior reversible encephalopathy syndrome*	7	36.8
Headache	5	71.4
Encephalopathy	2	28.6
Seizures	1	14.3
*Spinal cord involvement*	3	15.8
*Medulla oblongata involvement*	3	15.8
*Hypertrophic cranial pachymeningitis*	2	10.5
*Cerebellar ataxia*	2	10.5
*Non-CNS entities associated with CNS involvement*	13	68.4
Paranasal sinusitis	11	84.6
Autonomic nervous dysfunction	2	15.4
Orbital disease	1	7.7

CNS, Central Nervous System; EGPA, Eosinophilic Granulomatosis with Polyangiitis.

**Table 2:** Lesions characteristics, treatment and outcomes of EGPA patients with CNS involvement

ID	Sex/age	Lesions features	Lesions location	Induction therapy	Intrathecal injections^a^	FU, months	Outcomes
1	F/39	Ischemic infarctions	Multiple	MP 80 mg qd	N	1	death
2	F/46	Ischemic infarctions	Right semiovale centers	Pred 80 mg qd + CYC	N	84	remission^b^
3	M/49	Ischemic infarctions; PRES	Multiple, subacute cerebral infarcts in left basal ganglia	MP pulse + IVIG + CYC	DXM *3	72	remission
4	M/49	Ischemic infarctions	Basal ganglia	MP pulse	N	48	remission^b^
5	F/71	Spinal cord involvement	Thoracic spinal cord	MP 40 mg qd	N	24	remission
6	M/64	Ischemic infarctions; PRES	Right semiovale centers	MP 80 mg qd + IVIG + CYC	N	46	remission
7	M/52	Ischemic infarctions; PRES; ataxia;	Bilateral basal ganglia; cerebellar ataxia	MP 24 mg qd + CYC + AZA	DXM *1 + MTX *4	36	remission^b^
8	F/72	Ischemic infarctions	Multiple lesions in cerebrum	MP 160 mg qd	N	20	remission^b^
9	F/49	HCP	Cerebral falx	MP pulse	N	15	remission^b^
10	F/45	Ischemic infarctions; PRES	Under bilateral frontal and parieto-occipital cortex	Pred 40 mg qd + CYC	N	42	remission^b^
11	F/47	HCP	Bilateral brain	MP pulse	DXM *5	132	remission
12	M/57	Medulla oblongata and spinal cord lesions	Medulla oblongata and cervical spinal cord	MP pulse	N	17	remission
13	F/63	Ischemic infarctions	Right basal ganglia and white matter	MP pulse	N	12	remission
14	F/55	Intracranial hemorrhages; PRES	Right frontal–temporal lobe	MP pulse + Plasma change + CYC	N	1	death
15	M/46	Ischemic infarctions	Multiple lesions in cerebrum	MP 40 mg qd + CYC	N	4	remission
16	M/25	Medulla oblongata and spinal cord involvement	Medulla oblongata and thoracic spinal cord	Pred 17.5 mg qd + CYC	N	76	remission
17	M/34	Intracranial hemorrhages	Subarachnoid space	Pred 50 mg qd + CYC	N	9	remission^b^
18	M/41	Ischemic infarctions; PRES; medulla oblongata lesions; ataxia	Subcortex of bilateral frontal parietal lobe; right side of medulla oblongata; cerebellum	MP pulse + IVIG	N	20	remission
19	M/64	Ischemic infarctions; intracranial hemorrhages; PRES	Infarcts in cortex and white matter; cerebral hemorrhages in left basal ganglia, cortex, subcortex and the sulci	MP pulse	DXM *1	6	remission

Sex, F-female, M-male; FU, follow-up duration; MP, methylprednisolone; Pred, prednisone; IVIG, intravenous immunoglobulin; CYC, cyclophosphamide; PRES, posterior reversible encephalopathy syndrome; HCP, hypertrophic cranial pachymeningitis

^a^“N” indicates no intrathecal injections were used during the follow-up, and the number after the asterisk refers to the number of intrathecal injections in this column; DXM, Dexamethasone; MTX, methotrexate; AZA, azathioprine

^b^Relapse during glucocorticoid reduction;

**Fig. 1:**
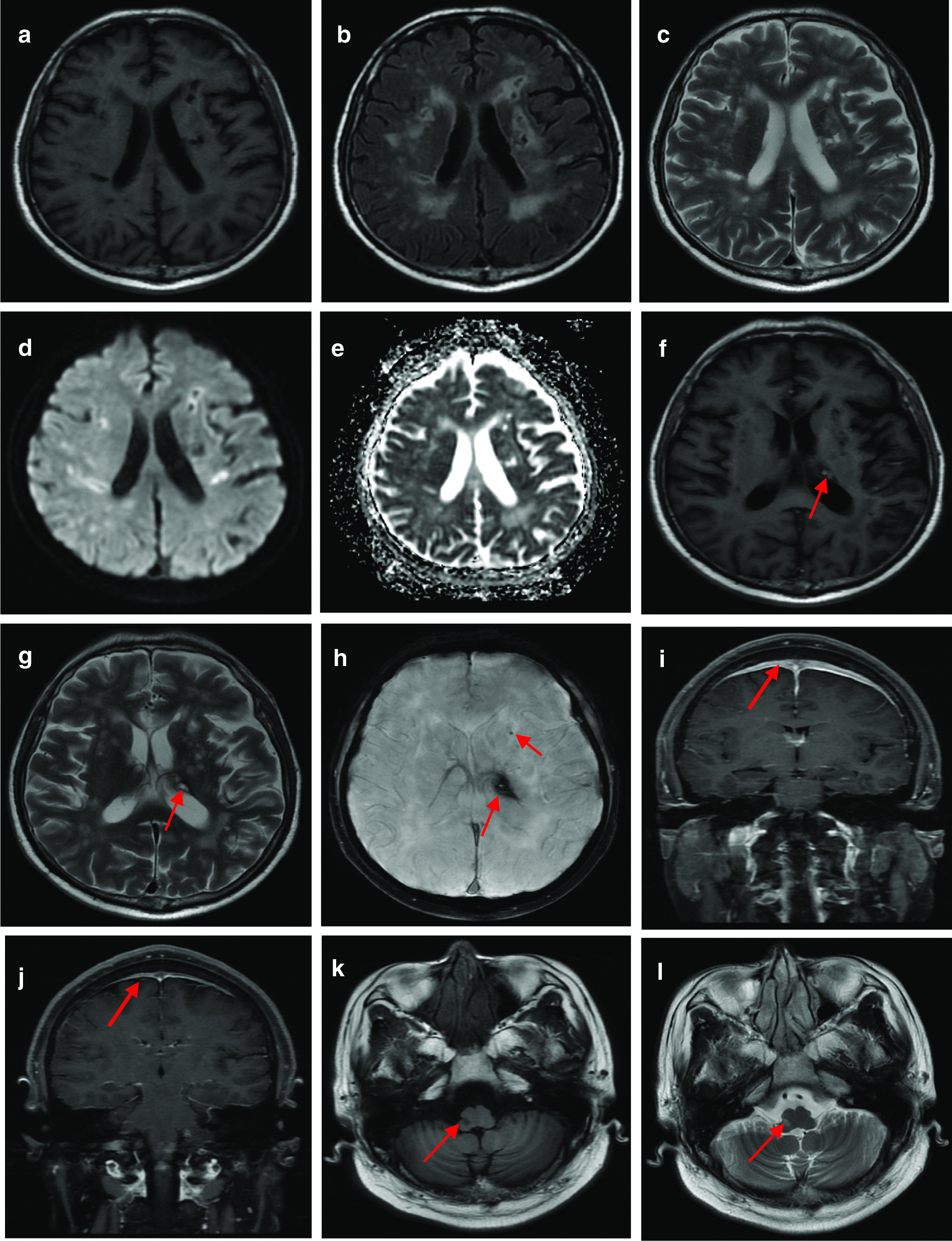
Brain magnetic resonance imaging of EGPA patients with central nervous system involvement. T1-weighted (**a**), T2 fluid-attenuated inversion recovery (Flair; **b**), T2 propeller (**c**), Diffusion-Weighted Images (**d**), and Apparent Diffusion Coefficient (**e**) show white matter lesions and multiple cerebral infarctions of Case 19; T1 Flair (**f**), T2 propeller (**g**) and T2*-weighted gradient-recalled echo (**h**) show intracerebral hemorrhages of Case 19. Another patient (Case 11) with hypertrophic cranial pachymeningitis (**i**) got alleviation of dural involvement after therapy (**j**). The images of T1 Flair (**k**) and T2 propeller (**l**) indicate the medulla oblongata involvement of one patient (Case 18). The corresponding lesions are indicated by the arrows.

Cerebrovascular events were the most common manifestations (73.7%), including twelve patients with ischemic lesions (85.7%) and three cases with intracranial hemorrhages (21.4%). Semiovale centers and basal ganglia were the most frequently affected location (7/14). Among the seven patients with posterior reversible encephalopathy syndrome (PRES), one patient experienced hoarseness, choking on swallowing, limb weakness, loss of consciousness, and sudden epilepsy. In three patients with spinal cord involvement, the thoracic cord was affected in two cases and the cervical cord in one case. Hypertrophic cranial pachymeningitis (HCP) mainly presented with a severe headache. Three patients had medulla oblongata involvement, one of whom had an absent pharyngeal reflex, and another patient complained of headache, convulsion, numbness and weakness, and dizziness. Figure 1 shows the representative magnetic resonance imaging (MRI).

Moreover, 13 cases (68.4%) were accompanied by non-CNS damage that was associated with CNS involvement in EGPA. Ethmoid sinusitis and maxillary sinusitis were found in 11 cases (84.6%), and autonomic nerves were affected in two cases (urinary and fecal incontinence).

### Baseline clinical characteristics of EGPA patients with or without CNS involvement

We compared the baseline clinical features between patients with CNS involvement and those without CNS involvement (Table 3). The mean age of EGPA onset in the case group was significantly older than that in the control group (51.7 ± 11.56 vs. 43.7 ± 13.78 years, *p* = 0.019). The disease duration in the case group was longer, which was close to the statistical difference [11 (4, 39) vs. 5 (2, 14) months, *p* = 0.082]. Regarding the clinical manifestations, the case group had a higher proportion of digestive tract involvement than the control group (52.6% vs. 28.6%, *p* = 0.042). And patients with CNS involvement showed an upward trend of fever incidence (57.9% vs. 37.4%, *p* = 0.098). The percentages of peripheral neuropathy in the two groups were comparable (57.9% vs. 44.0%, *p* = 0.268). Other organs, including the heart, lungs, kidneys, muscles, joints, skin, nose, and ears were found no statistical differences between the groups.

**Table 3:** Baseline features of EGPA patients with and without CNS involvement

Characteristics	With CNS involvement (n = 19)	Without CNS involvement (n = 91)	*P* value
*Demographics*			
Age (year)	51.7 ± 11.56	43.7 ± 13.78	0.019*
Gender (male/female, number)	10/9	51/40	0.490
Time from allergy to EGPA diagnosis (month)	48 (5, 115)	24 (5, 60)	0.314
Disease duration (month)	11 (4, 39)	5 (2, 14)	0.082
Time from initial symptoms to vasculitis (month)	21 (2, 54)	7 (0, 36)	0.337
*Clinical manifestation, n * (*%)*			
Weight loss	6 (31.6)	35 (38.5)	0.573
Fever	11 (57.9)	34 (37.4)	0.098
Arthritis	4 (21.1)	13 (14.3)	0.489
Myalgia	6 (31.6)	16 (17.6)	0.207
Allergic rhinitis	6 (31.6)	34 (37.4)	0.634
Severe asthma	13 (68.4)	72 (79.1)	0.368
Cutaneous vasculitis	11 (57.9)	48 (52.7)	0.682
Renal involvement	4 (21.1)	24 (26.4)	0.776
Digestive tract involvement	10 (52.6)	26 (28.6)	0.042*
Peripheral neuropathy	11 (57.9)	40 (44.0)	0.268
Cardiac involvement	5 (26.3)	35 (38.5)	0.317
Ear involvement	4 (21.1)	10 (11.0)	0.258
Sinusitis	11 (57.9)	53 (58.2)	0.978
*Laboratory examination*			
Eos count (10^9^/L)	4.1 (1.5,5.6)	2.6 (1.4,7.6)	0.770
Eos%	37.2 (11.8,50.0)	28.6 (17.1,45.7)	0.483
ESR (mm/1 h)	27 (9,40)	32 (14,54)	0.461
CRP (mg/L)	10.84 (5.34,39.77)	21.70 (4.54,63.85)	0.623
RF (IU/mL)	21.7 (7.0,90.1)	25.0 (10.0,90.4)	0.651
MPO-ANCA, n (%)	3 (15.8)	12 (13.2)	0.721
PR3-ANCA, n (%)	1 (5.3)	1 (1.1)	0.317
Biopsy-proven vasculitis	4 (21.1)	10 (11.0)	0.258
Granuloma, n (%)	1 (5.3)	5 (5.5)	1.000
*Clinical score*			
BVAS	16 (14,22)	14 (10,19)	0.103
FFS ≥ 1, n (%)	16 (84.2)	65 (71.4)	0.250

All the continuous normally distributed data are presented as mean ± standard deviation, and non-normally distributed data as median (first quartile, third quartile)

EGPA, Eosinophilic Granulomatosis with Polyangiitis; CNS, Central Nervous System; Eos, eosinophil; ESR, erythrocyte sedimentation rate; CRP, C-reactive protein; RF, rheumatoid factor; MPO, myeloperoxidase; ANCA, anti-neutrophil cytoplasmic antibody; PR3, protease 3; BVAS, Birmingham Vasculitis Activity Score; FFS, five -factor score; *P < 0.05.

### Independently associated factors for CNS involvement in EGPA patients

Next, we selected factors with two-sided *p* < 0.10 to explore independent factors associated with CNS involvement in EGPA by multivariate logistic regression analysis (Table 4). We found that age (odds ratio, OR = 1.045, 95% confidence interval, 95% CI 1.004–1.089), disease duration (OR = 1.018, 95% CI 1.001–1.036), and fever (OR = 3.134, 95% CI 1.007–9.755) were the potential independent risk factors for CNS involvement in EGPA.

**Table 4:** Multivariate analysis for 19 EGPA patients with CNS involvement and 91 controls

Variable	Odd Ratio	95% CI	*P* value
Age (years)	1.045	1.004–1.089	0.033*
Disease duration (months)	1.018	1.001–1.036	0.039*
Fever	3.134	1.007–9.755	0.049*
Digestive system involvement	1.906	0.637–5.706	0.249

EGPA, Eosinophilic Granulomatosis with Polyangiitis; CNS, Central Nervous System; CI, confidence interval; *P < 0.05.

### Features of the cerebrospinal fluid (CSF)

Seven patients underwent lumbar puncture, and four of them were found to have elevated intracranial pressure (245–300 mmH_2_O); one patient had bloody CSF due to subarachnoid hemorrhage. CSF protein was mildly increased in four patients (median 0.68 g/L), one of whom was accompanied by slightly raised CSF glucose (5.5 mmol/L).

### Treatment and outcomes

The treatment strategy for all EGPA patients with CNS involvement was summarized in Table 5. During the induction phase, glucocorticoids combined with cyclophosphamide (CYC) were used in 94.7% of all patients. Among them, nine patients (47.4%) were used methylprednisolone pulse (0.5–1.0 g/d, 3–5 days), nine patients (47.4%) were administered high-dose glucocorticoids (1–2 mg·kg^−1^·d^−1^), and only one patient (5.3%) was administered low-dose glucocorticoids (< 0.5 mg·kg^−1^·d^−1^) because of mild clinical symptoms. Compared with the control group, the case group had a higher ratio of methylprednisolone pulse (47.4% vs. 24.2%, *p* = 0.041). Additionally, three cases were administered intravenous immunoglobulin (IVIG), and one case was prescribed plasma exchange therapy. Notably, for those with severe CNS lesions (21.1%), we administered intrathecal injections of dexamethasone (10 mg each time) and methotrexate (MTX, 10 mg each time).

**Table 5:** Treatment and outcomes of EGPA patients with and without CNS involvement

	With CNS involvement (n = 19)	Without CNS involvement (n = 91)	*P* value
Treatment, n (%)			
GCs	19 (100)	88 (96.7)	1.000
MP pulse	9 (47.4)	22 (24.2)	0.041*
CYC	18 (94.7)	80 (87.9)	0.688
Rituximab	0 (0)	1 (1.1)	1.000
Plasma exchange	1 (5.3)	0 (0)	0.173
IVIG	3 (15.8)	11 (12.1)	0.706
Outcomes, n (%)			
Complete remission	17 (89.5)	73 (80.2)	0.517
Partial relief	0 (0)	12 (13.2)	0.122
Death	2 (10.5)	6 (6.6)	0.625

The categorical variables are expressed as frequencies and ratios, and are further compared by Chi-square analysis or Fisher’s exact test

EGPA, Eosinophilic Granulomatosis with Polyangiitis; CNS, Central Nervous System; GCs, glucocorticoids (including pulse, high, medium, and low doses); MP, methylprednisolone; CYC, cyclophosphamide (including all kinds of doses); IVIG, Intravenous immunoglobulin; *P< 0.05

In the maintenance treatment stage, low-dose glucocorticoids and the immunosuppressants, including MTX, Tripterygium wilfordii (used in traditional Chinese medicine), and azathioprine, were maintained according to the specific conditions for each patient.

**Fig. 2:**
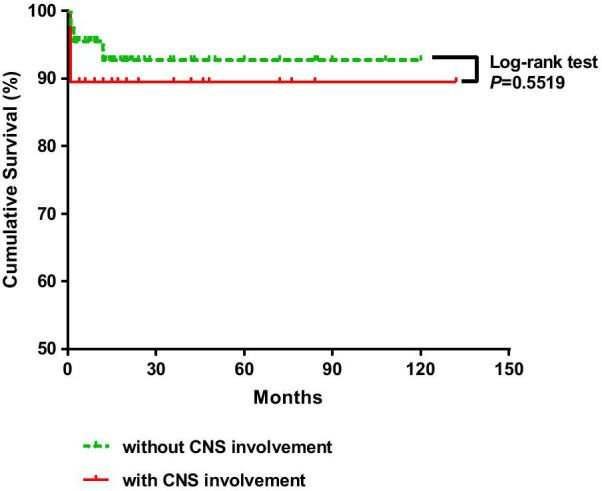
Comparison of cumulative survival rates between EGPA patients with and without central nervous system involvement

With a mean follow-up of 20 months (range 1–132 months), we witnessed that seventeen patients achieved clinical remission (89.5%), although seven of them relapsed during glucocorticoid reduction. Two patients died: one was due to an acute intracerebral hemorrhage within one month, and the other was due to gastrointestinal perforation. The cumulative survival (Fig. 2) and outcomes (Table 5) do not present significant differences between the groups.

## Discussion

In the single-center Chinese EGPA cohort, CNS involvement occurred in 17.3% of patients, which primarily manifested as ischemic lesions, followed by PRES, spinal cord, or medulla oblongata involvement. Age, disease duration, and fever were the independently associated factors for CNS lesions of EGPA. The treatment strategy was glucocorticoids-based immunosuppressive therapy, combined with CYC and intrathecal injections of dexamethasone and MTX.

CNS involvement in the current study was found in less than one-fifth of EGPA patients, suggesting that it was relatively rare compared to peripheral neuropathy in EGPA, which occurred in 46.4% of patients [11]. In studies from other countries, CNS lesions occurred in 5–14% of EGPA patients [7, 12–16], which was lower than that in our cohort. The reason may be that most of the patients admitted to our hospital were referred from the local hospitals with severe conditions. Similarly, CNS lesions were present in 7–16% of GPA [17–19] and 5.8–11.8% of MPA [17, 20, 21], indicating that all three types of AAV rarely affect the CNS.

The clinical manifestations of CNS involvement in EGPA vary widely, and all locations in the CNS could be affected, primarily including ischemic lesions, PRES, spinal cord or medulla oblongata involvement. This indicates the clinical identification complexity of CNS involvement in EGPA. Compared with EGPA, GPA is more prone to HCP [22, 23], and MPA is predominated by cerebrovascular neuropathy [17]. In EGPA, ischemic lesions were more commonly seen and mainly presented with multiple punctate ischemic foci or lacunar infarcts [10, 13]. In our cohort, subarachnoid hemorrhage occurred only in one patient, but previous studies reported a high proportion of subarachnoid hemorrhage [10], which may reflect the differences in ethnic populations and regions, or because our sample size was not large enough.

PRES is associated with autoimmune diseases, which are uncommon but unique in the late stages of AAV [24, 25]. PRES’ symptoms may relapse when the underlying EGPA is not well-controlled. Spinal cord damage usually affects the cervical and thoracic spinal cords, causing motor and sensory deficiencies. Medulla oblongata involvement should be noted considering the location’s vitality. Contrast-enhanced MRI helps capture abnormal signals in the spinal cord or medulla oblongata earlier.

Patients with CNS lesions are more likely to involve the digestive tract in our study. Age, disease duration, and fever were independently associated factors for CNS involvement in EGPA. Disease duration and fever reflect the inflammatory severity, which plays a pivotal role in CNS lesions, including innate and adaptive immunity and microbiota-gut-brain axis. The gastrointestinal tract is part of the largest immune cell compartments in the body. Therefore, the microbiota-gut-brain axis theory helps account for the association between the CNS and digestive tract involvement in EGPA [26]. Additionally, CNS-affected patients were older, which raises the possibility that age may affect the permeability of the small to medium-sized cerebral vessels and the severity of CNS vascular lesions in EGPA.

Patients with CNS involvement were prescribed more intense therapy, which mainly adopted methylprednisolone pulse or high-dose glucocorticoids combined with CYC. For severely ill patients, we specifically used intrathecal injections of dexamethasone and MTX, which contributed to a favorable clinical response. In 1994, Valesini and his colleagues reported for the first time the intrathecal treatment of central nervous system involvement in systemic lupus erythematosus [27]. Intrathecal injections of MTX 10 mg and dexamethasone 20 mg can bring dramatic therapeutic effects, and the symptoms of the nervous system can be controlled within 24 h. In China, especially in our hospital, this treatment strategy has been used since 1996 and shown good response to patients with CNS involvement of refractory lupus [28]. Gradually, the intrathecal treatment was also applied to CNS involvement of primary vasculitis. And in clinical practice, it has achieved a beneficial effect in some refractory EGPA patients with CNS involvement in China. In addition, high-dose IVIG has been demonstrated to be effective in patients who have failed in high-dose glucocorticoids induction therapy, as observed in our three patients with CNS involvement [29].

Although most patients achieved remission, about 40% of cases relapsed during glucocorticoid reduction, implying that better therapeutics are needed to replace glucocorticoids. Currently, only few types of biological agents have been tried in EGPA, including the B-cell antagonist monoclonal antibody Rituximab, the humanized monoclonal antibody against interleukin-5 Mepolizumab, and the recombinant humanized monoclonal anti-immunoglobulin E antibody Omalizumab. Firstly, for rituximab in China, the indications primarily include follicular B-cell non-Hodgkin's lymphoma, CD20-positive diffuse large B-cell non-Hodgkin's lymphoma. And the indications as off-label therapies include chronic graft-versus-host disease, thrombotic thrombocytopenic purpura, chronic lymphocytic leukemia, refractory severe systemic lupus erythematosus, rheumatoid arthritis, GPA, MPA. Therefore, rituximab used in EGPA was just based on the treatment experience of GPA and MPA. Secondly, compared with rituximab, CYC is more affordable, because rituximab is hundreds or even thousands of times more expensive than CYC. Moreover, CYC penetrates the blood–brain barrier more easily than rituximab [30]. Therefore, glucocorticoids combined with CYC are always the first-line therapy for CNS involvement of EGPA.

In recent reviews, rituximab was found it may help induce and sustain remission in patients with newly diagnosed or relapsing EGPA especially for ANCA-positive patients [31, 32]. But in the past, we did not find definite evidence for rituximab in CNS involvement of EGPA. And the ANCA-positive patients were relatively few in our cohort.

Other biological agents were more rarely used in EGPA due to a lack of evidence-based medicine data to prove their effectiveness for CNS involvement in EGPA. For example, mepolizumab was conformed effective on EGPA-related refractory asthma by randomized clinical trial [33–35], but no clinical trials were conducted for CNS involvement of EGPA. Besides, this biological agent has not yet been marketed in China.

The outcome analysis does not reveal statistical differences between the groups, possibly due to the following reasons: the sample size is not large enough for the nineteen patients with CNS involvement; the follow-up duration is relatively short, and observing sufficient adverse events may require longer follow-up duration. Other studies observed that long-term neurological sequelae were common in CNS involved EGPA patients, occurring in at least half of patients, and were mainly associated with cerebrovascular events [10].

There are limitations to this study. First, this is a single-center retrospective study, and CNS involvement in EGPA is very rare. Thus, the sample size is not large enough, and we could not evaluate the prevalence of CNS involvement in EGPA accurately. Second, most of the patients included in the cohort primarily had worse conditions who were referred from the local hospitals. Therefore, they could not represent a larger EGPA population in China. Thus, we need to enroll more patients with EGPA and continue to follow up to observe the impact of CNS involvement on the long-term survival of EGPA patients.

## Conclusions

CNS involvement is uncommon, occurring in 17.3% of EGPA patients. Its manifestations vary widely, including cerebrovascular events, PRES, spinal cord or medulla oblongata involvement, HCP, and cerebellar ataxia. Ischemic lesions are the most frequent manifestations. Age, disease duration, and fever are independent factors associated with CNS involvement in EGPA. After timely treatment of glucocorticoids combined with CYC and intrathecal injections, most patients achieve clinical remission. Acute intracranial hemorrhage and gastrointestinal perforation may be the principal causes of death.

## Methods

### Patients

We conducted a single-center retrospective case–control study and included 110 EGPA patients from Peking Union Medical College Hospital (PUMCH) between January 2007 and March 2019. This cohort had 19 EGPA patients with CNS involvement as the case group, and 91 EGPA patients without CNS involvement as the control group. All patients fulfilled the 2012 Revised International Chapel Hill Consensus Conference Nomenclature of Vasculitides criteria or the American College of Rheumatology 1990 criteria, and the diagnosis of EGPA was verified by two rheumatologists [36, 37].

For EGPA patients who may have CNS involvement, we systematically evaluated the central nervous system conditions according to their clinical manifestations, physical examinations, MRI and computed tomography scan of the brain or spinal cord, and laboratory testing of CSF. Firstly, we ruled out the CNS lesions caused by other CNS disease-related risk factors, including hypertension, hyperlipidemia, hyperglycemia, smoking, infection, and genetic factors. According to the literature and the recommendation of neurologists, we diagnosed the new onset or relapsing EGPA based on extra-CNS features of EGPA. Concomitantly the manifestations of CNS involvement occurred and responded to immunosuppressive therapy, which were defined as EGPA-related CNS lesions [22].

Manifestations of CNS involvement in EGPA had cerebrovascular events (including ischemic lesions and intracranial hemorrhages), PRES, spinal cord or medulla oblongata involvement, HCP, and cerebellar ataxia. EGPA-related PRES refers to a clinic radiological disorder of reversible vasogenic brain edema in patients with classic acute neurological symptoms, which mainly present with seizures, encephalopathy, headache, and visual disturbances in the setting of EGPA and exclude other possible causes [38, 39]. HCP was diagnosed by contrast-enhanced MRI or computed tomography scan of the brain. Non-CNS entities associated with CNS involvement were analyzed because their anatomical locations were close, and often coincided with CNS involvement.

Considering that this study was based on a retrospective design and that we only used the medical records that did not involve any private information of patients and would not harm them, written informed consent was not given, but oral informed consent was obtained from all enrolled patients by face to face or phone. Additionally, when each patient was admitted to our hospital, our doctors stated that their clinical data might be used for scientific research, and all patients signed the written informed consent at that time. Furthermore, this study was approved by the Medical Ethics Committee of PUMCH (Beijing, China, approval number: S-K1385).

### Clinical and laboratory assessments

CSF analysis included pressure (100–180 mmH2O, for adults), appearance (colorless and transparent fluid), protein (0.15–0.45 g/L), and glucose (2.8–4.5 mmol/L). For all EGPA patients, we examined each system included in the Birmingham Vasculitis Activity Score (BVAS) to analyze disease activity [40]. Severe asthma was defined as requiring continuous glucocorticoid therapy (a high dose of inhaled, oral, or intravenous glucocorticoids) or with persistent dyspnea. Cutaneous vasculitis included palpable purpura, reticulata, and gangrene ischemia of the extremities. The definition of arthritis was the symptoms of swelling and pain in multiple joints, accompanied by morning stiffness. Renal involvement was defined as the presence of active urinary sediment (hematuria or cylindruria), proteinuria (urine protein > 0.5 g/24 h), or serum creatinine beyond the upper limit of the normal range. Cardiac involvement included pericardial effusion, myocardial involvement, heart failure, coronary lesions, moderate to severe valve involvement, and arrhythmia, which cannot be explained by other reasons after consultation with experienced cardiologists. Digestive system involvement was defined as gastrointestinal bleeding, intestinal obstruction, or digestive tract conditions that other mechanisms could not explain. Peripheral neuropathy included mononeuritis multiplex and multiple peripheral neuropathy (mononeuritis multiplex meant two or more separate non-adjacent nerve trunks were simultaneously or successively involved, and multiple peripheral neuropathy predominantly affected the distal extremities in a bilaterally symmetrical distribution).

The 2009 revised FFS system [9] and the original 1994 BVAS [40] were used to evaluate the prognosis and disease activity of vasculitis at diagnosis, respectively. The remissions included complete remission and partial relief, which were defined as a BVAS of 0 and a 50% or more decrease in BVAS compared to the baseline, respectively. The death referred to all-cause mortality.

### Statistical analysis

Data were described as mean ± standard deviation (SD) for continuous normally distributed data or median (first quartile, third quartile) for continuous non-normally distributed data. And they were compared using the Student's *t*-test and Mann–Whitney *U* test, respectively. Categorical variables were expressed as frequencies and ratios, which were further compared by Chi-square analysis or Fisher’s exact test. Factors with two-sided *P* < 0.10 were included in the multivariate logistic regression analysis, which was presented with OR and 95% CI. Since FFS includes the factors of age and severe gastrointestinal involvement, we excluded FFS in this model to avoid duplication. Kaplan–Meier survival curves and log-rank tests were used to illustrate and compare the cumulative survival rates (Prism 7; GraphPad, San Diego, CA, USA). A two-sided *P* value < 0.05 was considered a statistically significant difference. Statistical analysis was performed using SPSS (Statistical Product and Service Solutions; International Business Machines Corporation25, Armonk, NY, USA).

## Data Availability

The relevant data used to support the findings of the study are included in the article.

## Abbreviations

EGPA: Eosinophilic granulomatosis with polyangiitis
AAV: Antineutrophil cytoplasmic antibody-associated vasculitis
CNS: Central nervous system
PUMCH: Peking Union Medical College Hospital
BVAS: Birmingham Vasculitis Activity Score
FFS: Five-factor score
CSF: Cerebrospinal fluid
MRI: Magnetic resonance imaging
SD: Standard deviation
OR: Odds ratio
CI: Confidence interval
CYC: Cyclophosphamide
MTX: Methotrexate
IVIG: Intravenous immunoglobulin
GPA: Granulomatosis with polyangiitis
MPA: Microscopic polyangiitis
HCP: Hypertrophic cranial pachymeningitis
PRES: Posterior reversible encephalopathy syndrome
